# Skin prick testing does not reflect the presence of IgE against food allergens in adult eosinophilic esophagitis patients: a case study

**DOI:** 10.1186/1476-7961-8-16

**Published:** 2010-11-17

**Authors:** Toral A Kamdar, Anne M Ditto, Paul J Bryce

**Affiliations:** 1 Division of Allergy-Immunology, Feinberg School of Medicine, Northwestern University, Chicago, IL 60610, USA

## Abstract

Skin prick testing is widely used to predict the presence of allergen-specific IgE. In eosinophilic esophagitis patients, who frequently exhibit polysensitization and broad reactivity upon skin prick testing, this is commonly used to aid avoidance recommendations in the clinical management of their disease. We present here the predictive value of skin prick testing for the presence of allergen-specific IgE, in 12 patients, determined by immunoblot against the allergen extracts using individual-matched serum. Our results demonstrate a high degree of predictive value for aeroallergens but a poor predictive value for food allergens. This suggests that skin prick testing likely identifies IgE reactivity towards aeroallergens in adult eosinophilic esophagitis but this is not true for foods. Consequently, IgE immunoblotting might be required for determining food avoidance in these patients.

## Background

Eosinophilic esophagitis (EoE) is a gastrointestinal disorder that is associated with allergic disease. Studies have described personal or family histories of asthma or allergic rhinitis in adult EoE patients [1,2] and a murine model has shown that aeroallergen sensitization may be responsible for the eosinophilic response in the esophagus [3]. Adult EoE patients display a broad range of reactivity to multiple allergens that spans both aero and food allergens. In one study, 81% of EoE patients had poly-reactivity to environmental allergens on skin prick testing (SPT) [4]. In light of such broad reactivity, we queried the reliability of SPT for determining actual IgE-mediated reactions in this patient population.

## Case Study

Here, we compared the presence of specific IgE (determined by Western blots) to SPT in patients with biopsy-proven EoE. Blood was drawn from 12 EoE patients (demographics, EoE diagnostic criteria, allergic status and therapeutic treatments shown in Table 1) who were SPT positive (wheal size greater than 3 mm with surrounding erythema) to a minimum of one aero and one food allergen. None of the patients has a history of IgE-mediated food allergy and, instead had been determined as EoE patients due to a history of dysphagia, presence of endoscopic characteristics (e.g. rings, furrows, strictures) and pathological assessment of eosinophils in esophageal biopsy tissue that were greater than 25 per high powered field (hpf). Western blot-based screening for IgE-specific recognition of proteins in 5 aero and 5 food allergen extracts was undertaken and compared to the SPT outcomes towards the same extracts. In determining the positive or negative responses by Western blot, each extract was resolved, transferred and probed with patient specific serum. The presence of IgE specific to bands within the extracts was determined using HRP-labeled anti-human IgE. While several bands were observed, the results reflect the presence or absence of any recognition.

**Table 1:** Patient demographics, allergic status and treatments.

Patient	Age	Gender	Allergic Rhinitis	Asthma	Eos per HPF	Elimination Diet	Swallowed steroids
*A*	48	M	Yes	No	50	Yes	No
*B*	43	M	Yes	Yes	>25	No	No
*C*	71	M	Yes	Yes	>25	Yes	No
*D*	34	M	Yes	No	>25	No	No
*E*	33	F	Yes	Yes	>25	Yes	No
*F*	39	F	Yes	No	>150	No	Yes
*G*	47	F	Yes	Yes	>25	No	Yes
*H*	42	F	Yes	Yes	>25	No	No
*I*	36	M	Yes	No	>25	No	Yes
*J*	51	F	Yes	Yes	>25	Yes	Yes
*K*	56	F	Yes	No	>25	Yes	No
*L*	30	M	Yes	Yes	>25	Yes	Yes
*M*	32	M	Yes	No	>25	No	Yes
*N*	38	M	Yes	No	>25	No	No
*O*	39	F	Yes	No	>25	No	No

The overall match comparison between SPT and immunoblot for all allergens was 75.6% (Table 2). However, aeroallergens vastly outweighed food allergens in their SPT reliability. The combined reliability for aeroallergens was 89% while foods were only 56%. The positive predictive value of skin testing for aeroallergens was 95%, with only 3 false positives; all were to maple. Cat was responsible for most false negatives (3/12). Conversely, food allergen SPT correlated poorly with immunoblot reactivity, with false negatives common for wheat and whole milk (6/12 and 7/12) and false positives for peanut (2/12). In all cases, patients displayed negative reactivity for some of the extracts, indicating the specificity of the immunoblot approach for determining positive or negative IgE binding to the specific allergen extracts.

**Table 2:** Results of SPT versus IgE-specific recognition by Western Blot

Allergen	**Positive/Positive**^**1**^	Positive/Negative	Negative/Positive	Negative/Negative
Cat	7	0	3	2
Bermuda grass	11	0	0	1
Blue grass	10	0	1	1
Dust mite	11	0	1	0
Ragweed	12	0	0	0
Maple tree	7	3	2	0

Wheat	5	0	7	0
Whole milk	4	0	6	2
Shrimp^2^	1	0	0	0
Peanut	5	2	0	5
Almond^3^	7	0	3	1
Whole egg	1	4	2	5

^1. Refers to number of skin tests positive/number of western blots positive^

^2. Only one patient had skin testing done to shrimp^

^3. Only eleven patients had skin testing done to almond^

Consequently, SPT for adult EoE patients may be a reliable method for determining IgE-associated reactivity towards aeroallergens. However, while patients demonstrated the presence of food protein-specific serum IgE, SPT appears inadequate for determining this. Alternatively, EoE patients may be refractory to food specific IgE triggering by SPT via an unknown mechanism.

## Conclusions

Clinically, while elemental diet-therapy has been attempted for EoE, it is often not well-tolerated in adults due to its stringency and limitations. Instead, specific food elimination (SFE) has been explored, the foods for which are generally determined by SPT. Various studies have reported SPE as less efficacious than elemental diets [5]. This could be partially explained by poor correlation of SPT with actual IgE-specific reactivity. A report in pediatric EoE, suggested to contain both IgE and non-IgE reactions, concluded that a combination of SPT with atopic patch test (to identify the non-IgE reaction) might be beneficial for determining foods to eliminate for SPE [6]. Our data supports previous findings [4] that adult EoE patients are highly IgE positive but now suggests that SPT may actually fail to identify the presence of food allergen-specific IgE and that immunoblot be required to determine this. Alternatively, Immuno-CAP based determination could be useful is assessing the quantitative IgE levels, although the actual quantities of food allergen specific IgE are generally not thought to correlate with disease severity [7].

In conclusion, our data is the first study to determine the reliability of SPT as a detection method for determining the presence of allergen-specific IgE within adult EoE patients. Within this patient group, aeroallergen reactivity by SPT faithfully predicts the presence of specific IgE, despite such broad reactivity. Conversely, food allergens exhibit both false negatives and false positives that diminish confidence in the SPT response for concluding IgE presence. As a consequence, direct determination of food allergen-specific IgE may be beneficial in identifying the likely food triggers for clinicians considering food elimination therapy in EoE patients.

## Consent

Written informed consent was obtained from all patients to allow materials to be used for research purposes and subsequent reports, under protocols approved by the Northwestern University Institutional Review Board.

## List of abbreviations used

EOE: Eosinophilic esophagitis; HPF: High Power Field; IGE: Immunoglobulin E; SPE: Specific Food Elimination diet; SPT: Skin prick test.

## Competing interests

The authors declare that they have no competing interests.

## Authors' contributions

TK, AD, PB designed the experiments. TK, AD obtained samples. TK, PB performed the experiments. TK, PB wrote the manuscript. All authors have read and approved this work.

## References

[B1] Arora AS Yamazaki K Eosinophilic esophagitis: asthma of the esophagus? Clin Gastroenterol Hepatol 2004 2 523 530 10.1016/S1542-3565(04)00236-8 15224275

[B2] Simon D Marti H Heer P Simon HU Braathen LR Straumann A Eosinophilic esophagitis is frequently associated with IgE-mediated allergic airway diseases J Allergy Clin Immunol 2005 115 1090 1092 10.1016/j.jaci.2005.01.017 15867873

[B3] Mishra A Hogan SP Brandt EB Rothenberg ME An etiological role for aeroallergens and eosinophils in experimental esophagitis J Clin Invest 2001 107 83 90 10.1172/JCI10224 11134183 PMC198543

[B4] Roy-Ghanta S Larosa DF Katzka DA Atopic characteristics of adult patients with eosinophilic esophagitis Clin Gastroenterol Hepatol 2008 6 531 535 10.1016/j.cgh.2007.12.045 18304887

[B5] Spergel JM Eosinophilic esophagitis in adults and children: evidence for a food allergy component in many patients Current opinion in allergy and clinical immunology 2007 7 274 278 10.1097/ACI.0b013e32813aee4a 17489048

[B6] Spergel JM Brown-Whitehorn T Beausoleil JL Shuker M Liacouras CA Predictive values for skin prick test and atopy patch test for eosinophilic esophagitis J Allergy Clin Immunol 2007 119 509 511 10.1016/j.jaci.2006.11.016 17291865

[B7] Sicherer SH Sampson HA Peanut allergy: emerging concepts and approaches for an apparent epidemic J Allergy Clin Immunol 2007 120 491 503 quiz 504-495 10.1016/j.jaci.2007.07.015 17689596

